# Supplementary material for: Using QC-Blind for Quality Control and Contamination Screening of Bacteria DNA Sequencing Data Without Reference Genome
Source: Front Microbiol. 2019 Jul 9;10:1560. doi: 10.3389/fmicb.2019.01560 (PMC6637319; doi:10.3389/fmicb.2019.01560)
Supplement: Supplementary File 2 — (Table S1) Identification Sensitivity of target and contamination species for QC-Blind-processed simulated datasets. (Table S2) Assembly Statistics for simulated datasets. [file Data_Sheet_2.docx]

**Additional File 2**

**Table S1 | Identification Sensitivity of target and contamination species for QC-Blind-processed simulated datasets.**

|  | **Sensitivity** | **Species** | **Genus** | **Family** |
| --- | --- | --- | --- | --- |
| **Simu_BS_5%** | target sensitivity | 0% | 100% | 100% |
| **Simu_BS_35%** | target sensitivity | 0% | 100% | 100% |
| **Simu_BS_65%** | target sensitivity | 0% | 100% | 100% |
| **Simu_BS_95%** | target sensitivity | 0% | 100% | 100% |
|  | Average | 0% | 100% | 100% |
| **Simu_BS_5%** | contamination sensitivity | 20% | 90% | 100% |
| **Simu_BS_35%** | contamination sensitivity | 10% | 90% | 100% |
| **Simu_BS_65%** | contamination sensitivity | 20% | 80% | 100% |
| **Simu_BS_95%** | contamination sensitivity | 10% | 90% | 90% |
|  | Average | 15% | 88% | 98% |

Identification of target and contamination at family, genus and species level are shown in this table.

**Table S2 | Assembly Statistics for simulated datasets.**

|  | **BS 5%** | **BS 35%** | **BS 65%** | **BS 95%** |
| --- | --- | --- | --- | --- |
| **Velvet N50** | 39369 | 49287 | 47030 | 24697 |
| **Velvet #contig** | 10667 | 6193 | 6232 | 11150 |
| **MEGAHIT N50** | 154200 | 154200 | 154200 | 27129 |
| **MEGAHIT #contig** | 603 | 620 | 599 | 4135 |

N50 and number of contigs were derived from Velvet and MEGAHIT in simulated data.
